# Life expectancy and healthy life expectancy of patients with advanced schistosomiasis in Hunan Province, China

**DOI:** 10.1186/s40249-023-01053-8

**Published:** 2023-01-28

**Authors:** Honglin Jiang, Jie Zhou, Meng Xia, Guangping Li, Jie Di, Feng Mao, Liangqing Yu, Yu Cai, Zhengzhong Wang, Ying Xiong, Yixin Tong, Jiangfan Yin, Yue Chen, Qingwu Jiang, Yibiao Zhou

**Affiliations:** 1 Fudan University School of Public Health, Building 8, 130 Dong’an Road, Shanghai, 200032 China; 2 Key Laboratory of Public Health Safety, Fudan University, Ministry of Education, Building 8, 130 Dong’an Road, Shanghai, 200032 China; 3 Fudan University Center for Tropical Disease Research, Building 8, 130 Dong’an Road, Shanghai, 200032 China; 4 Hunan Institute for Schistosomiasis Control, Jin’e Middle Road, Yueyang, 414021 Hunan China; 5 Yueyang Vocational and Technical College, Xueyuan Road, Yueyang, 414000 Hunan China; 6 Hunan Institute Xiangyue Hospital, Jin’e Middle Road, Yueyang, 414022 Hunan China; 7 School of Epidemiology and Public Health, Faculty of Medicine, University of Ottawa, 600 Peter Morand Crescent, Ottawa, ON K1G 5Z3 Canada

**Keywords:** Advanced schistosomiasis, Life expectancy, Healthy life expectancy, Mortality, Medical assistance

## Abstract

**Background:**

Few studies have investigated the change in life expectancy (LE) and the healthy lifespan among patients with advanced schistosomiasis. This study was to evaluate the LE and healthy life expectancy (HLE) for patients and assess the mechanism responsible for the LE inequality.

**Methods:**

We utilized data from a dynamic advanced schistosomiasis cohort (10,362 patients) for the period from January 2008 to December 2019 in Hunan Province, China, to calculate the LEs of patients, and made a comparison with that of general population (19,642 schistosomiasis-free individuals) in the schistosomiasis endemic areas. LEs were estimated from 15 years of age by constructing period life tables. Arriaga’s decomposition method was applied to quantify the influence of the age structure on the difference in LE. HLE for advanced schistosomiasis patients was calculated by using Sullivan method with age-specific disability weight. The LE and HLE were calculated for both males and females to perform further analyses on gender gap.

**Results:**

The estimated LE for advanced schistosomiasis patients aged 15–19 was 49.51 years (48.86 years for males and 51.07 years for females), which was 20.14 years lower compared with general population (69.65 years), and the LE gap between patients and general population decreased with age. The largest age-specific mortality contribution to the gap (32.06%) occurred at age 80–84 years. Women had a lower LE and HLE than men at age ≥ 60 years (both gender gaps in LE and HLE < 0). For advanced schistosomiasis patients, the gender gap in LE was largely attributed to the difference in mortality among those under the age of 55; the age-specific mortality in women exerted positive influence on the gap at age 25–64 and 75–79 years, with the contribution rate ranging from 0.59% to 57.02%, and made the negative contribution at other age groups.

**Conclusions:**

The LE of advanced schistosomiasis patients was still much lower compared with general population. Strengthened prevention strategies and targeted treatments are needed to reduce morbidity and mortality due to advanced schistosomiasis, especially for younger population and elderly female patients.

**Supplementary Information:**

The online version contains supplementary material available at 10.1186/s40249-023-01053-8.

## Background

Advanced schistosomiasis is the most severe form of schistosomiasis japonica, a serious neglected tropical disease with considerable morbidity endemic in China, Indonesia, and the Philippines [1–3]. Lack of timely and effective treatment or long-term repeated infections can lead chronic schistosomiasis to advanced schistosomiasis [3], and cause sustained damage to both intestine and liver parenchyma, bleeding of the upper gastrointestinal tract, spontaneous bacterial peritonitis, hepatic failure, and a high level of mortality or disability [4–6]. In the past, the mean survival of the advanced patients in China was only 5.16 years [7], and the rate of potential years of life lost of all causes of death was 25.63% [8].

The incidence and prevalence of schistosomiasis have been steadily decreasing [9], and patients with advanced schistosomiasis have been experiencing lengthening lifespan in China during the past several decades due to effective schistosomiasis control measures [10]. In 2005, China launched a national medical assistance program in which large funds are expended by the government to assist patients with advanced schistosomiasis in treatment, and achieved a great reduction in the mortality [11, 12]. However, there are still around 30,000 patients with advanced schistosomiasis in the country [13], and few studies have investigated their current lifespan and change in life expectancy (LE).

As an important summary measure of mortality, LE reflects the health conditions of all age groups and covers multiple health dimensions, and is therefore crucial for exploring the health status of a specific population and for strengthening health policy decisions [14]. Yu et al. assessed the LE of patients with advanced schistosomiasis in 1992 and found that the LE was 26.80 years at 10 years of age and was more than 30 years lower compared with the general population [15]. The LE has not been reevaluated since then. Large inequalities in the LE between two populations may exist since the age-specific mortality rates vary substantially [16]. Decomposition of the contributions of age-specific mortality change could reveal which age groups are mainly responsible for the change in LE, and thus help with more targeted therapeutic efforts for individuals.

Notably, increased longevity is not simply equivalent to increased quality of life. Severe disability caused by advanced schistosomiasis (e.g., anaemia, growth retardation) also leads to a heavy disease burden for patients [17]. A recent study showed an upward trend for the disease burden of advanced schistosomiasis [18]. The growth of advanced schistosomiasis cases and disease burden implies an increasing demand for health care and medical support. Healthy life expectancy (HLE) is a comprehensive indicator, which derives its change from changes in both mortality and health status [19]. It could thus reflect the health trends of advanced schistosomiasis patients and provide the implication for medical interventions in improving patients’ quality of life.

To fill the knowledge gap in LE and to better understand the current health status for advanced schistosomiasis, we evaluated the LE and HLE of patients and made a comparison to the general population from schistosomiasis endemic areas in Hunan Province, China. We also explored the mechanism of gender inequality in LE through decomposition analysis by age groups.

## Methods

### Data sources

We first utilized data from a dynamic advanced schistosomiasis cohort in Hunan Province, China from January 2008 to December 2019. The cohort was established in 2005 by the Hunan Institute for Schistosomiasis Control (HISC), and has thereafter continuously recruited advanced schistosomiasis patients who are registered in the National Medical Assistance Program [20]. HISC collects the patients’ baseline information (e.g., demographics, epidemiological history, and treatment history) and obtains written informed consent from patients themselves or their close relatives at enrollment. Clinical data and outcomes are acquired from each observation of admission or annual follow-up. Details of the data collection have been previously described [4]. As patients with advanced schistosomiasis mainly died from schistosomiasis and its related complications, the death outcome that appeared in this study was regarded as all-cause death.

We then obtained population and mortality data for general population from 2019 census in 11 national schistosomiasis surveillance sites with severe epidemic in Hunan Province. Schistosomiasis surveillance sites, established in villages, refer to areas endemic with schistosomiasis where routine surveillance and emergency endemic surveillance are implemented with main efforts on case reports, case surveys and acute schistosomiasis warnings [21, 22]. Therefore, community dwelling members without *S. japonica* infection in these areas can be a representative sample of general population comparable to those infected. Information on the age, sex, and death date of deceased persons was collected. Totally, 10,362 advanced schistosomiasis patients and 19,642 schistosomiasis-free individuals were included in this study.

### Statistical analysis

#### Calculation of LE

LEs were calculated with the construction of period life tables based on an average of age-specific death rates for a 5-year time period [23]. For both patients with advanced schistosomiasis and general population, LEs were estimated from 15 years of age since there were no patients under age 15. For patients with advanced schistosomiasis, we calculated age-specific mortality rates based on the numbers of observed person-years and deaths for each age group during 2008–2019 and then converted them into life table age-specific probabilities of dying. For general population, the age-specific mortality rates were calculated for the year of 2019. A probability of dying of 1 was assigned to the open-ended interval age group (≥ 85 years in this study). Other life table functions (e.g., probability of surviving, number of years lived and total number of years lived) and LE were defined and computed using the standard life table expressions [23]. Life tables were also constructed for males and females separately, and the decomposition of age-specific mortality contribution to the gender gap in LE was examined. The loss years of LE due to advanced schistosomiasis was the difference in LE between the patients and general population, and the loss rate was calculated as lost years of LE divided by LE of general population.

#### Decomposition of the LE gap

Among various decomposition approaches [24–26], Arriaga's method [24] was applied for its easy application to life table data and the capability to provide effects of the last open-ended age group. We estimated the age-specific mortality contributions to: (i) the LE difference between general population and patients with advanced schistosomiasis overall as well as for males and females separately; (ii) the gender gap in LE among both patient and general groups. The method measures the LE gap and decomposes the overall change into three components with additivity: a direct effect, an indirect effect, and an interaction effect [27]. The direct effect on the difference in LE results from mortality changes within each age group, whereas the indirect effect is due to a difference in the number of survivors at the end of that age interval from a mortality change within a specific age group. The interaction effect reflects the impact combining the changed number of survivors at the end of the age interval and the lower (or higher) mortality rates at older ages [24, 27].

The formula consists of two mathematical terms (i.e., direct effect, indirect and interaction effects):

$${}_{n}T{E}_{x}=\left[\frac{{l}_{x}^{1}}{{l}_{0}}\times \left(\frac{{L}_{x}^{2}}{{l}_{x}^{2}}-\frac{{L}_{x}^{1}}{{l}_{x}^{1}}\right)\right]+\left[\frac{{T}_{x+n}^{2}}{{l}_{0}}\times \left(\frac{{l}_{x}^{1}}{{l}_{x}^{2}}-\frac{{l}_{x+n}^{1}}{{l}_{x+n}^{2}}\right)\right].$$

For the last open-ended age interval, the contribution can be expressed as:

$${TE}_{x}^{^{\prime}}=\frac{{l}_{x}^{1}}{{l}_{0}}\times \left(\frac{{T}_{x}^{2}}{{l}_{x}^{2}}-\frac{{T}_{x}^{1}}{{l}_{x}^{1}}\right),$$

where _*n*_*TE*_*x*_ is the total contribution between age *x* and *x* + *n*, *l*_*x*_ and *l*_*x*+*n*_ is the number of individuals alive at age *x* and *x* + *n*, *l*_*0*_ is the hypothetical cohort size (commonly 100,000), *L*_*x*_ is the number of person-years lived within the single-age interval, and *T*_*x*_ and *T*_*x*+*n*_ is the total number of person-years lived above age *x* and *x* + *n*. "1" represents the reference group (e.g., general population; male group), and "2" represents advanced schistosomiasis patients or female groups.

Another decomposition method proposed by Pollard [25] was also used to compare with the results from Arriaga's method, and checked for their consistency.

#### Calculation of HLE

Sullivan method [28] was applied to calculate the HLE in patients with advanced schistosomiasis within each 5-year age interval based on life tables and disability data of patients [19]. This method is widely used in the estimation of HLE for its straightforward and succinctness [29–31]. In this study, HLE represents the schistosomiasis-free life expectancy for advanced cases. We applied the age-specific disability weight (DW) of advanced schistosomiasis japonica proposed by Jia et al. [32] to transform the health status of patients and obtain the hypothetical number of surviving person-years without disability. The DW is 0.510 for age ≥ 60 years, 0.399 for age 45–59 years, and 0.378 for age < 45 years, estimated based on patients' self-rated health scores on the visual analogue scale of an “EQ-5D plus” questionnaire [32].

The HLE is calculated as follows:

$${HLE}_{x}=\frac{1}{{l}_{x}}\times \sum_{i=x}^{w}\left[{L}_{x}\times \left(1-{D}_{x}\right)\right].$$

The corresponding loss of HLE is calculated as:

$${LHE}_{x}=\frac{1}{{l}_{x}}\times \sum_{i=x}^{w}\left({L}_{x}\times {D}_{x}\right),$$

where *HLE*_*x*_ and *LHE*_*x*_ are the HLE and HLE loss years for advanced schistosomiasis patients respectively, *w* represents the largest age category, *l*_*x*_ is the number of survivors at age *x*, *L*_*x*_ is the total number of person-years lived above age *x* and *x* + *n*, and *D*_*x*_ is the DW for individuals within an age group (*x*, *x* + *n*). Subgroup analysis was done by sex to examine the gender inequality in HLE. The left of healthy years (i.e., HLE left) within a specific age group was obtained by calculating the difference of HLE between age *x* and age *x* + *n*.

This study was approved by the Ethical Review Committee of the School of Public Health, Fudan University, China. All data analyzed was fully anonymized.

## Results

### LEs for advanced schistosomiasis patients and general population

**Fig. 1:**
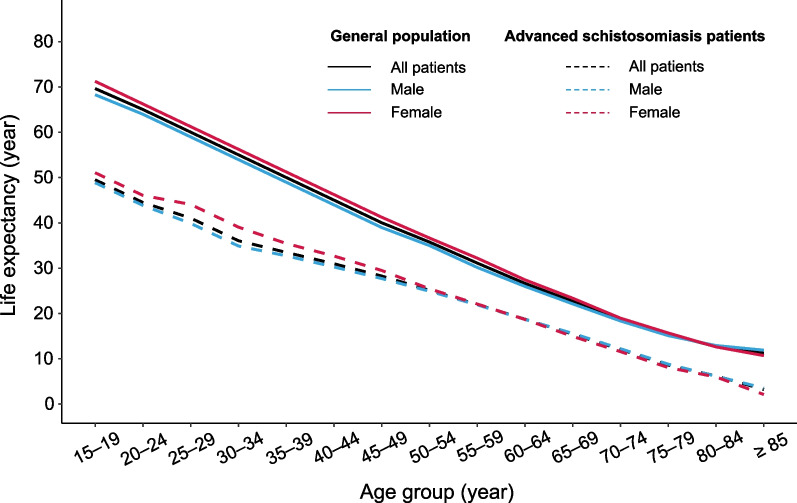
Life expectancy in patients with advanced schistosomiasis and general population

**Fig. 2:**
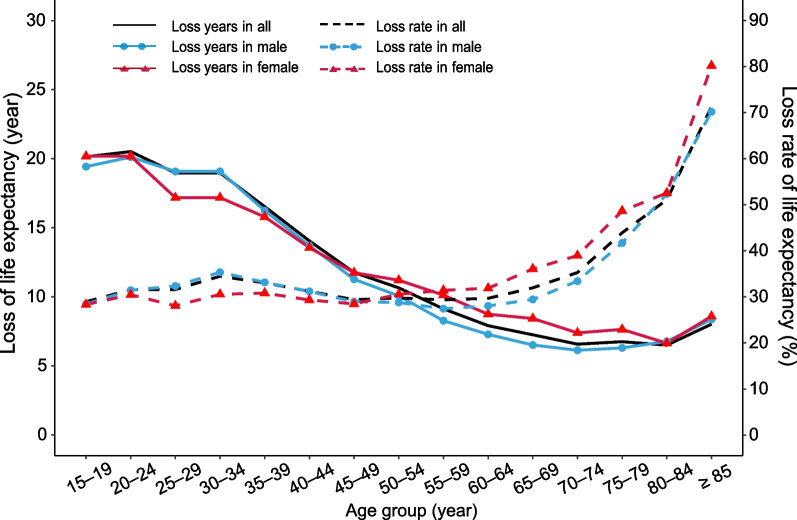
The loss years and loss rates of life expectancy in patients with advanced schistosomiasis

The estimated LE at age group 15–19 was 49.51 years for patients with advanced schistosomiasis (48.86 years for males and 51.07 years for females), which was 20.14 years lower than that of general population (69.65 years) as shown in Table 1. The LE gap between the patients and schistosomiasis-free people tended to decrease with age regardless of gender. For both groups, the LE for females was higher at younger age (gender gap > 0) but lower at older age (gender gap < 0) compared with males (Table 1; Fig. 1), while the opposite was true for the loss of LE (Fig. 2). The gender gap in LE was more pronounced at younger age for both groups; the LE for women descended faster than men among patients, with a greater lost rate of LE after age 50 (Fig. 2). Details in LE for both patients and general population are presented in Additional file 1.

**Table 1:** The life expectancy of advanced schistosomiasis patients and general population

Age group	Advanced schistosomiasis patients (a)				General population (b)				LE gap between two groups (b–a)		
	Total	Male	Female	Gender gap	Total	Male	Female	Gender gap	Total	Male	Female
15–19	49.51	48.86	51.07	2.21	69.65	68.29	71.25	2.96	20.14	19.43	20.18
20–24	44.51	43.86	46.07	2.21	65.03	63.98	66.25	2.27	20.52	20.12	20.18
25–29	41.06	39.90	44.07	4.17	60.03	58.98	61.25	2.27	18.97	19.08	17.18
30–34	36.06	34.90	39.07	4.17	55.03	53.98	56.25	2.27	18.97	19.08	17.18
35–39	33.50	32.74	35.46	2.72	50.03	48.98	51.25	2.27	16.53	16.24	15.79
40–44	30.98	30.27	32.69	2.42	45.03	43.98	46.25	2.27	14.05	13.71	13.56
45–49	28.27	27.71	29.50	1.79	40.03	38.98	41.25	2.27	11.76	11.27	11.75
50–54	25.12	24.92	25.46	0.54	35.77	34.99	36.66	1.67	10.65	10.07	11.20
55–59	22.00	21.90	22.10	0.20	31.13	30.17	32.22	2.05	9.13	8.27	10.12
60–64	18.74	18.70	18.69	− 0.01	26.65	25.98	27.43	1.45	7.91	7.28	8.74
65–69	15.44	15.61	14.94	− 0.67	22.69	22.12	23.37	1.25	7.25	6.51	8.43
70–74	12.06	12.22	11.58	− 0.64	18.63	18.35	18.97	0.62	6.57	6.13	7.39
75–79	8.62	8.81	8.06	− 0.75	15.37	15.11	15.70	0.59	6.75	6.30	7.64
80–84	6.22	6.16	5.99	− 0.17	12.72	12.93	12.63	− 0.30	6.50	6.77	6.64
≥ 85	3.22	3.55	2.12	− 1.43	11.24	11.91	10.71	− 1.20	8.02	8.36	8.59

Gender gap, calculated as the LE of females minus that of males within each age group

*LE* life expectancy

### Decomposition of age-specific mortality contribution

**Fig. 3:**
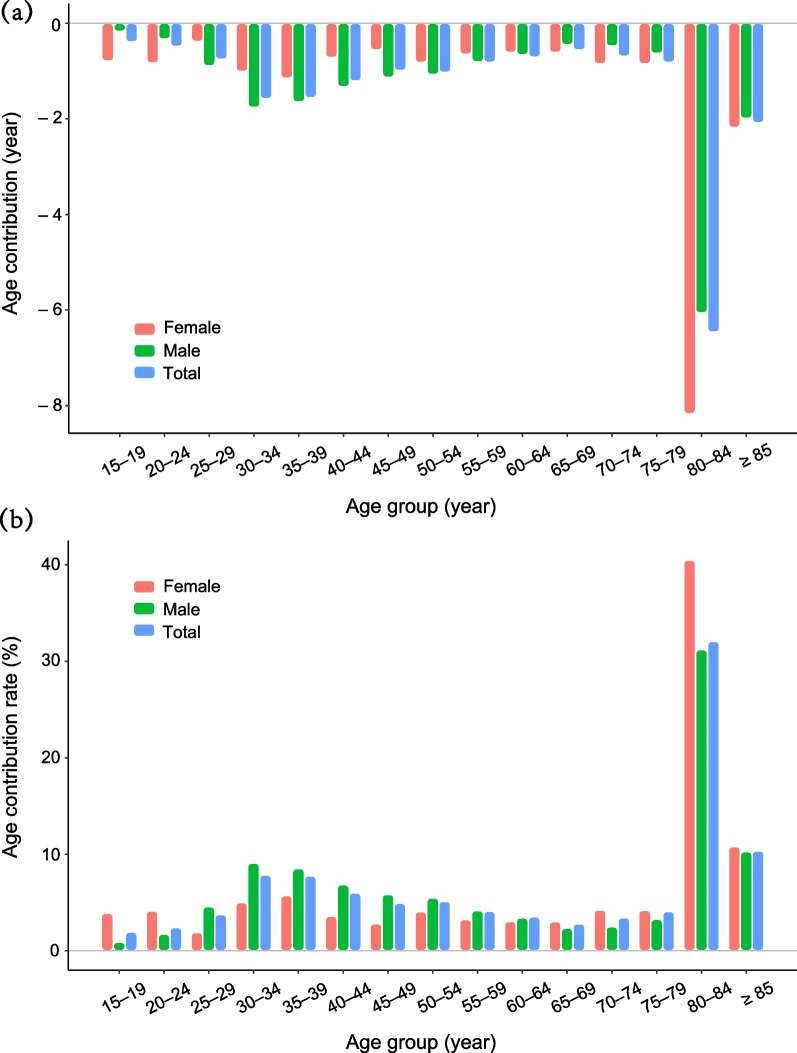
Age-specific mortality contribution to the gap in LE between advanced schistosomiasis patients and general population. **a** Age-specific mortality contribution to the gap in LE (years). **b** The rate of age-specific mortality contribution to the gap in LE. *LE* life expectancy

**Fig. 4:**
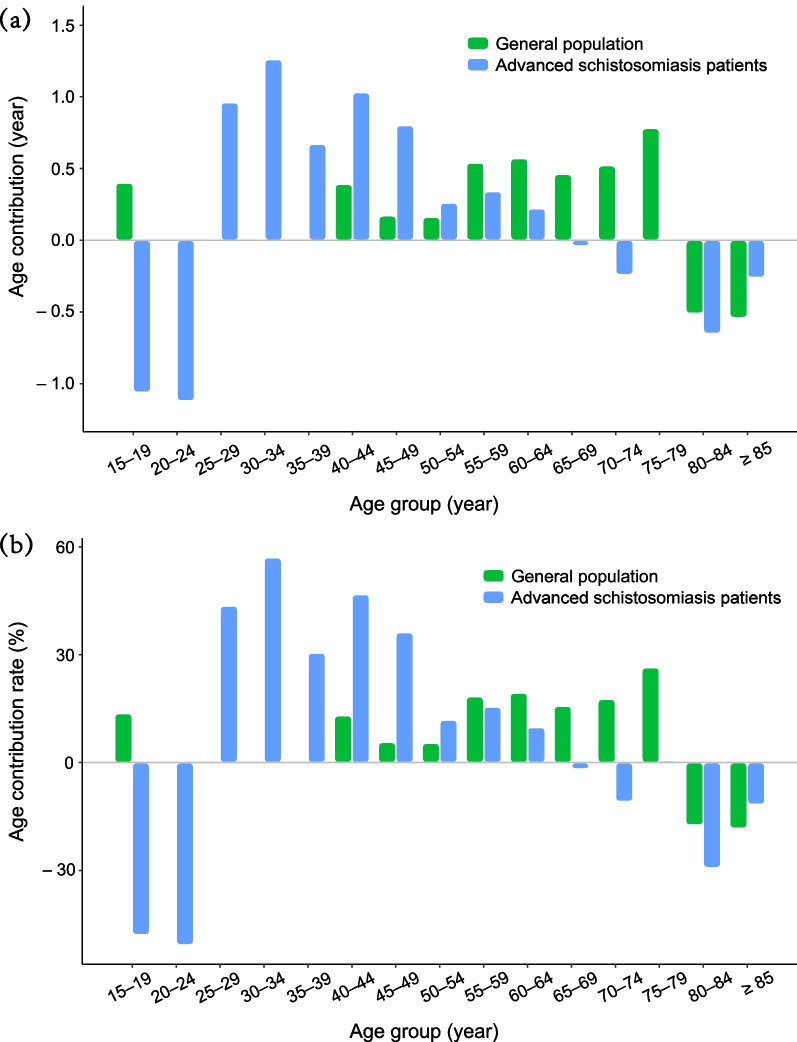
Age-specific mortality contribution to the gender gap in LE among advanced schistosomiasis patients. **a** Age-specific mortality contribution to the gender gap in LE (years). **b** The rate of age-specific mortality contribution to the gender gap in LE. *LE* life expectancy

Results of age-specific mortality contributions calculated by Arriaga's method are shown in Figs. 3 and 4. For both males and females, higher mortality rates in patients with advanced schistosomiasis made the largest contribution to the decline in LE at age 80–84 years (Fig. 3a), accounting for nearly one third of the loss (6.46 years (32.06%) for all patients, 6.06 years (31.21%) for males, and 8.17 years (40.48%) for females) (Fig. 3b). Under the age of less than 80, the contribution of changes in age-specific mortality ranged from an increase (contribution rate: from 0.89% to 9.07% in those aged 15–34 years) to a decrease (contribution rate: from 8.51% to 2.33% in those aged 35–69 years) for males, while there was no clear pattern in age-specific mortality contribution for females. Compared with males, females showed a larger negative impact of the mortality change on the reduction of LE at age of < 25 (contribution rate: female 3.89–4.12% vs male 0.89–1.70%) or ≥ 65 (contribution rate: female 3.01–40.48% vs male 2.33–31.21%) than other age groups (Additional file 2).

The contribution of age-specific mortality change to the LE difference between males and females are presented in Fig. 4 and Additional file 3. The age contribution pattern of advanced schistosomiasis patients was different from that of general population. Under age of 55, the age-specific mortality contribution to gender inequality in LE was more pronounced for patients but less pronounced for general population, as the absolute contribution rate of all age groups before 55 was larger in patients (11.86–57.02%) than in general population (0.07–13.66%).

For advanced schisotosomiasis patients, the change in age-specific mortality in those aged 25–64 and 75–79 years contributed positively (increased LE > 0) to the gender gap in LE (Fig. 4a), with the contribution rate ranging from 0.59% to 57.02% (Fig. 4b; Additional file 3). Such contribution pattern of age-specific mortality difference in gender was much dissimilar to that of general population, but shifted to the same negative impact (increased LE < 0) in older groups aged above 80, indicating a substantial influence of disease on the survival for young and middle-age people with advanced schistosomiasis.

Decomposed by Pollard’s method, however, the advanced-age (80–84 years) mortality contributed much smaller (an overall 0.53 years (2.75%) of LE loss) than most of other age groups, due probably to the increasing residual effects in the advanced age groups. Although lacking estimation from the open-ended age interval, results of Pollard’s decomposition showed a similar contribution pattern to that of Arriaga's method in age groups of 30–69 (Additional file 4). Given the overt deviation of the LE difference produced by Pollard’s method, results from Arriaga's method might be more precise in current data and were subsequently discussed.

### HLE for advanced schistosomiasis patients

**Fig. 5:**
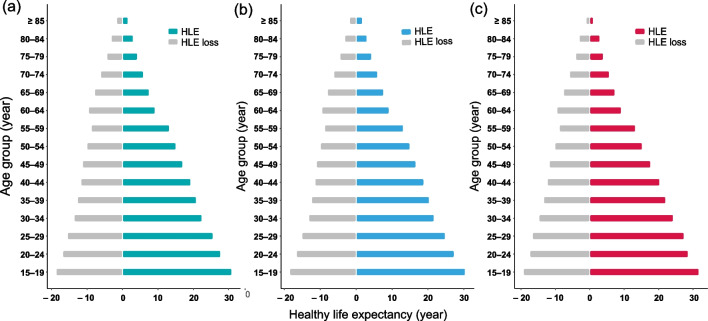
Healthy life expectancy and the loss of healthy years of patients with advanced schistosomiasis. **a** All patients. **b** Males. **c** Females. *HLE* healthy life expectancy

The HLE of advanced schistosomiasis patients at age 15–19 were 30.80 years for all the patients, 30.39 years for males, and 31.76 years for females (Fig. 5; Additional file 5). Overall, the loss years of HLE decreased with age. Patients tended to lost less HLE at the age of 55–59 (8.78 years), but later experienced a greater decrease in HLE at age 60–64 years (9.56 years). There was a consistent trajectory of the gender gap for HLE and LE in advanced schistosomiasis patients (Additional file 5). Females lost less HLE (gender gap > 0) in younger ages but more (gender gap < 0) in older ages (≥ 60) compared to males, with a decreasing gap from age 35 to 64.

## Discussion

This study examined the LE and HLE among patients with advanced schistosomiasis in Hunan province, China and made a comparison with general population. The study demonstrated that both the reduction of LE due to advanced schistosomiasis and the gender inequality in LE varied with age-specific mortality. Our findings have strong practical implications on clinical treatment for advanced schistosomiasis; also, they could provide useful information for more effective implementation of medical assistance policies and allocation of health resources in the future.

LE has significantly increased for advanced schistosomiasis patients (from 24.95 at age 15 in 1992 [15] to 49.51 years at present). Our study showed that patients with advanced schistosomiasis still had a 20-year lower LE compared with general population. The change in mortality due to advanced schistosomiasis for the very elderly (≥ 80 years) mainly contributed to the loss of LE, indicating a prolonged lifespan in advanced schistosomiasis patients. Apart from the long disease course from chronic to late stage resulting in high morbidity in older population [33], the implementation of medical support helped reduce the mortality rate of complications related to advanced schistosomiasis and improve patients’ health conditions [11].

Despite extended lifespan, patients with advanced schistosomiasis lost more than 18 years of HLE. Most patients would be accompanied by heavy disability and experience poor quality of life due to decreased aerobic capacity and complications relevant to impaired liver function [4, 32]. A few studies attempted to assess the patient's overall health status from the perspective of physical conditions combined with working capacity and psychological fitness [5, 34, 35], but none revealed how long the patients could live with a healthy life. We evaluated patients’ potential healthy lifespan based on the age-specific DW, and found that women had a lower HLE and a higher loss rate of HLE than men after the age of 60. This result indicated that improved interventions including treatment and medical care may be required for elderly women.

The gender inequity in LE varied considerably with age, and our study showed a similar picture for general population as compared with previous studies conducted in China and other countries [36–38], where the primary contributions to gender gap were derived from advanced age groups. For advanced schistosomiasis patients, the gender gap in LE was largely attributed to the significant change in mortality at younger ages (mainly < 55 years), indicating a more profound impact of the disease on the gender inequality in survival probability of younger patients. Such contrary findings might be explained by lifestyle risk factors [39, 40] that are associated with unfavorite prognosis of chronic liver damages. In addition, our study indicated that males at young and middle ages (25–59 years) were more vulnerable to advanced schistosomiasis-related death than female counterparts. The reason for the higher mortality of middle-age males is likely related to individual behaviors, such as smoking and alcohol consumption which were demonstrated to drive hepatic fibrosis progression [41, 42] and might thus increase liver tissue injury induced by schistosome infection [43]; however, it needs further studies to confirm. It highlighted the importance of early detection, diagnosis, and treatment of schistosome infection to prevent or delay the progression to advanced stage.

This study provided new estimates of LE and HLE for advanced schistosomiasis patients. Major strengths include the prospective nature of study design (dynamic cohort), large sample size (all patients in Hunan province during a 11-year period), rarely lost to follow-up (medical assistance program), and stringent mortality ascertainment. There are also some limitations. First, there was no information on specific causes of death to perform decomposition analysis, and the results could be biased towards the impact of advanced schistosomiasis on LE and overestimate in HLE. Second, there was a lack of information on the influencing factors of LE associated with advanced schistosomiasis (e.g., degree of infection, lifestyles, severity of disease, and treatment history), and further research is needed to interpret the LE inequality among different populations. Third, we only used age-specific DW to calculated HLE. When available, DW estimates based on individuals’ symptoms could be applied to refine the gender difference in HLE. Fourth, validation of LE in patients from other schistosomiasis epidemic regions is needed for comparison.

## Conclusions

This study estimated the current LE and HLE of advanced schistosomiasis in Hunan Province, China. The LE for patients significantly improved but was still much lower compared with general population. Women had lower LE and HLE and a high loss rate than men at advanced ages. The age-specific mortality contributions to the gender gap in LE were centralized in young and middle-age among patients with advanced schistosomiasis. Our findings highlighted the necessity of strengthening prevention strategies to reduce morbidity and premature deaths due to advanced schistosomiasis at younger ages, and targeting actions of health improvement for elderly female patients. Financial and technological support in treatment remains much to desire so that the medical assistance project could be assessible to more patients.

## Supplementary Information

**Additional file 1**: Life expectancies of patients with advanced schistosomiasis and general population.

**Additional file 2**: Decomposition of the age-specific mortality contribution to the gap in life expectancy between advanced schistosomiasis patients and general population.

**Additional file 3**: Decomposition of the age-specific mortality contribution to the gender gap in life expectancy of advanced schistosomiasis and general population groups.

**Additional file 4: **Age-specific contributions to the changes in life expectancy decomposed by Pollard’s decomposition method.

**Additional file 5:** Healthy life expectancy and its gender gap among patients with advanced schistosomiasis.

## Data Availability

The data that support the findings of this study are available from Hunan Institute for Schistosomiasis Control, Hunan, China, but restrictions apply to the availability of these data, which were used under license for the current study, and so are not publicly available. Data are however available from the authors upon reasonable request and with permission of Hunan Institute for Schistosomiasis Control, Hunan, China.

## Abbreviations

LE: Life expectancy
HLE: Healthy life expectancy
HISC: Hunan Institute for Schistosomiasis Control
DW: Disability weight
