# Supplementary material for: Life expectancy and healthy life expectancy of patients with advanced schistosomiasis in Hunan Province, China
Source: Infect Dis Poverty. 2023 Jan 28;12:4. doi: 10.1186/s40249-023-01053-8 (PMC9883924; doi:10.1186/s40249-023-01053-8)
Supplement: Supplementary file 1 — Additional file 1: Life expectancies of patients with advanced schistosomiasis and general population. [file 40249_2023_1053_MOESM1_ESM.docx]

**Life expectancies of patients with advanced schistosomiasis and general population**

| **Patients with advanced schistosomiasis (2008-2019)** | | | | | | | | | | | | | | |
| --- | --- | --- | --- | --- | --- | --- | --- | --- | --- | --- | --- | --- | --- | --- |
| **Age group** | **Interval** | | **All patients（n=10362）** | | | | **Male（n=7307）** | | | | **Female（n=3055）** | | | |
|  |  | | **Observed personal year** | | **Observed death** | **LE** | **Observed personal year** | | **Observed death** | **LE** | **Observed personal year** | | **Observed death** | **LE** |
| 15–19 | 5 | | 62.25 | | 0 | 49.51 | 51.33 | | 0 | 48.86 | 10.92 | | 0 | 51.07 |
| 20–24 | 5 | | 275.42 | | 2 | 44.51 | 200.33 | | 1 | 43.86 | 75.08 | | 1 | 46.07 |
| 25–29 | 5 | | 467.83 | | 0 | 41.06 | 323.33 | | 0 | 39.90 | 144.50 | | 0 | 44.07 |
| 30–34 | 5 | | 1355.67 | | 19 | 36.06 | 954.75 | | 16 | 34.90 | 400.92 | | 3 | 39.07 |
| 35–39 | 5 | | 3834.50 | | 59 | 33.50 | 2917.58 | | 47 | 32.74 | 916.92 | | 12 | 35.46 |
| 40–44 | 5 | | 6866.25 | | 106 | 30.98 | 5057.08 | | 85 | 30.27 | 1809.17 | | 21 | 32.69 |
| 45–49 | 5 | | 7146.83 | | 99 | 28.27 | 5004.75 | | 84 | 27.71 | 2142.08 | | 15 | 29.50 |
| 50–54 | 5 | | 10183.75 | | 163 | 25.12 | 7209.25 | | 122 | 24.92 | 2974.50 | | 41 | 25.46 |
| 55–59 | 5 | | 14216.58 | | 243 | 22.00 | 9669.67 | | 172 | 21.90 | 4546.92 | | 71 | 22.10 |
| 60–64 | 5 | | 13618.83 | | 270 | 18.74 | 9181.08 | | 204 | 18.70 | 4437.75 | | 66 | 18.69 |
| 65–69 | 5 | | 11464.50 | | 270 | 15.44 | 8224.25 | | 190 | 15.61 | 3240.25 | | 80 | 14.94 |
| 70–74 | 5 | | 7461.33 | | 226 | 12.06 | 5537.92 | | 168 | 12.22 | 1923.42 | | 58 | 11.58 |
| 75–79 | 5 | | 2500.38 | | 175 | 8.62 | 1990.50 | | 125 | 8.81 | 599.88 | | 50 | 8.06 |
| 80–84 | 5 | | 672.48 | | 57 | 6.22 | 457.88 | | 45 | 6.16 | 214.60 | | 12 | 5.99 |
| ≥ 85 | 5 | | 70.93 | | 22 | 3.22 | 60.30 | | 17 | 3.55 | 10.63 | | 5 | 2.12 |
| **General population (2019)** | | | | | | | | | | | | | | |
| **Age group** | | **Interval** | | **Total population（n=19642）** | | | **Male（n=10133）** | | | | **Female（n=9509）** | | | |
|  | |  | | **Number** | **Death** | **LE** | **Number** | **Death** | | **LE** | **Number** | **Death** | | **LE** |
| 0–1 | | 1 | | 38 | 0 | 84.65 | 18 | 0 | | 83.29 | 20 | 0 | | 86.25 |
| 1–4 | | 4 | | 418 | 0 | 83.65 | 224 | 0 | | 82.29 | 194 | 0 | | 85.25 |
| 5–9 | | 5 | | 812 | 0 | 79.65 | 430 | 0 | | 78.29 | 382 | 0 | | 81.25 |
| 10–14 | | 5 | | 906 | 0 | 74.65 | 499 | 0 | | 73.29 | 407 | 0 | | 76.25 |
| 15–19 | | 5 | | 885 | 1 | 69.65 | 476 | 1 | | 68.29 | 409 | 0 | | 71.25 |
| 20–24 | | 5 | | 934 | 0 | 65.03 | 506 | 0 | | 63.98 | 428 | 0 | | 66.25 |
| 25–29 | | 5 | | 1461 | 0 | 60.03 | 735 | 0 | | 58.98 | 726 | 0 | | 61.25 |
| 30–34 | | 5 | | 1907 | 0 | 55.03 | 945 | 0 | | 53.98 | 962 | 0 | | 56.25 |
| 35–39 | | 5 | | 1342 | 0 | 50.03 | 687 | 0 | | 48.98 | 655 | 0 | | 51.25 |
| 40–44 | | 5 | | 1531 | 0 | 45.03 | 804 | 0 | | 43.98 | 727 | 0 | | 46.25 |
| 45–49 | | 5 | | 2040 | 8 | 40.03 | 1095 | 6 | | 38.98 | 945 | 2 | | 41.25 |
| 50–54 | | 5 | | 1875 | 4 | 35.77 | 948 | 1 | | 34.99 | 927 | 3 | | 36.66 |
| 55–59 | | 5 | | 1381 | 5 | 31.13 | 694 | 4 | | 30.17 | 687 | 1 | | 32.22 |
| 60–64 | | 5 | | 1068 | 9 | 26.65 | 527 | 5 | | 25.98 | 541 | 4 | | 27.43 |
| 65–69 | | 5 | | 1107 | 10 | 22.69 | 572 | 7 | | 22.12 | 535 | 3 | | 23.37 |
| 70–74 | | 5 | | 730 | 15 | 18.63 | 381 | 8 | | 18.35 | 349 | 7 | | 18.97 |
| 75–79 | | 5 | | 569 | 19 | 15.37 | 274 | 11 | | 15.11 | 295 | 8 | | 15.70 |
| 80–84 | | 5 | | 357 | 21 | 12.72 | 187 | 12 | | 12.93 | 170 | 9 | | 12.63 |
| ≥ 85 | | 5 | | 281 | 25 | 11.24 | 131 | 11 | | 11.91 | 150 | 14 | | 10.71 |

LE, life expectancy.
