# Supplementary material for: Life expectancy and healthy life expectancy of patients with advanced schistosomiasis in Hunan Province, China
Source: Infect Dis Poverty. 2023 Jan 28;12:4. doi: 10.1186/s40249-023-01053-8 (PMC9883924; doi:10.1186/s40249-023-01053-8)
Supplement: Supplementary file 2 — Additional file 2: Decomposition of the age-specific mortality contribution to the gap in life expectancy between advanced schistosomiasis patients and general population. [file 40249_2023_1053_MOESM2_ESM.docx]

**Decomposition of the age-specific mortality contribution to the gap in life expectancy between advanced schistosomiasis patients and general population***

| **Age group** | **Total population** | | | | |  | **Male** | | | | |  | **Female** | | | | |
| --- | --- | --- | --- | --- | --- | --- | --- | --- | --- | --- | --- | --- | --- | --- | --- | --- | --- |
|  | **Direct effect** | **Indirect effect** | **Total effect** | **Contribution rate (%)** | **Increased LE (y)** |  | **Direct effect** | **Indirect effect** | **Total effect** | **Contribution rate (%)** | **Increased LE (y)** |  | **Direct effect** | **Indirect effect** | **Total effect** | **Contribution rate (%)** | **Increased LE (y)** |
| 15–19 | 0.01 | -0.75 | -0.74 | 1.94 | -0.39 |  | 0.03 | -0.35 | -0.32 | 0.89 | -0.17 |  | 0.00 | -1.70 | -1.70 | 3.89 | -0.79 |
| 20–24 | -0.09 | -0.83 | -0.92 | 2.42 | -0.49 |  | -0.06 | -0.56 | -0.62 | 1.70 | -0.33 |  | -0.16 | -1.64 | -1.80 | 4.12 | -0.83 |
| 25–29 | 0.00 | -1.43 | -1.43 | 3.75 | -0.75 |  | 0.00 | -1.65 | -1.65 | 4.57 | -0.89 |  | 0.00 | -0.82 | -0.82 | 1.89 | -0.38 |
| 30–34 | -0.17 | -2.83 | -3.00 | 7.85 | -1.58 |  | -0.20 | -3.09 | -3.29 | 9.07 | -1.76 |  | -0.09 | -2.09 | -2.18 | 4.99 | -1.01 |
| 35–39 | -0.18 | -2.77 | -2.96 | 7.75 | -1.56 |  | -0.19 | -2.89 | -3.08 | 8.51 | -1.65 |  | -0.16 | -2.34 | -2.50 | 5.72 | -1.15 |
| 40–44 | -0.19 | -2.10 | -2.29 | 5.99 | -1.21 |  | -0.20 | -2.28 | -2.48 | 6.84 | -1.33 |  | -0.14 | -1.43 | -1.57 | 3.59 | -0.72 |
| 45–49 | -0.12 | -1.76 | -1.88 | 4.92 | -0.99 |  | -0.13 | -1.98 | -2.11 | 5.83 | -1.13 |  | -0.06 | -1.16 | -1.22 | 2.80 | -0.56 |
| 50–54 | -0.16 | -1.79 | -1.95 | 5.12 | -1.03 |  | -0.18 | -1.80 | -1.99 | 5.48 | -1.07 |  | -0.13 | -1.64 | -1.77 | 4.05 | -0.82 |
| 55–59 | -0.15 | -1.41 | -1.56 | 4.09 | -0.82 |  | -0.14 | -1.37 | -1.51 | 4.16 | -0.81 |  | -0.17 | -1.24 | -1.41 | 3.23 | -0.65 |
| 60–64 | -0.13 | -1.22 | -1.34 | 3.51 | -0.71 |  | -0.14 | -1.09 | -1.23 | 3.40 | -0.66 |  | -0.09 | -1.24 | -1.33 | 3.03 | -0.61 |
| 65–69 | -0.15 | -0.91 | -1.06 | 2.78 | -0.56 |  | -0.11 | -0.73 | -0.85 | 2.33 | -0.45 |  | -0.21 | -1.11 | -1.32 | 3.01 | -0.61 |
| 70–74 | -0.09 | -1.21 | -1.30 | 3.42 | -0.69 |  | -0.09 | -0.81 | -0.90 | 2.48 | -0.48 |  | -0.10 | -1.75 | -1.85 | 4.23 | -0.85 |
| 75–79 | -0.28 | -1.27 | -1.55 | 4.07 | -0.82 |  | -0.17 | -1.01 | -1.18 | 3.26 | -0.63 |  | -0.45 | -1.39 | -1.83 | 4.19 | -0.85 |
| 80–84 | -0.16 | -12.09 | -12.24 | 32.06 | -6.46 |  | -0.18 | -11.12 | -11.30 | 31.21 | -6.06 |  | -0.02 | -17.66 | -17.68 | 40.48 | -8.17 |
| ≥ 85 | -3.95 | 0.00 | -3.95 | 10.35 | -2.08 |  | -3.72 | 0.00 | -3.72 | 10.26 | -1.99 |  | -4.71 | 0.00 | -4.71 | 10.78 | -2.18 |
| Total | -5.80 | -32.37 | -38.17 | 100.00 | -20.14 |  | -5.48 | -30.74 | -36.22 | 100.00 | -19.43 |  | -6.47 | -37.20 | -43.67 | 100.00 | -20.18 |

LE, life expectancy.

* By applying Arriaga's decomposition method.
