# Supplementary material for: Life expectancy and healthy life expectancy of patients with advanced schistosomiasis in Hunan Province, China
Source: Infect Dis Poverty. 2023 Jan 28;12:4. doi: 10.1186/s40249-023-01053-8 (PMC9883924; doi:10.1186/s40249-023-01053-8)
Supplement: Supplementary file 3 — Additional file 3: Decomposition of the age-specific mortality contribution to the gender gap in life expectancy of advanced schistosomiasis and general population groups. [file 40249_2023_1053_MOESM3_ESM.docx]

**Decomposition of the age-specific mortality contribution to the gender gap in life expectancy of advanced schistosomiasis and general population groups***

| **Age group** | **Advanced schistosomiasis patients** | | | | |  | **General population** | | | | |
| --- | --- | --- | --- | --- | --- | --- | --- | --- | --- | --- | --- |
|  | **Direct effect** | **Indirect effect** | **Total effect** | **Contribution rate (%)** | **Increased LE (y)** |  | **Direct effect** | **Indirect effect** | **Total effect** | **Contribution rate (%)** | **Increased LE (y)** |
| 15–19 | 0.00 | -1.05 | -1.05 | -47.83 | -1.06 |  | 0.03 | 0.37 | 0.40 | 13.66 | 0.40 |
| 20–24 | -0.10 | -1.01 | -1.11 | -50.65 | -1.12 |  | 0.00 | 0.00 | 0.00 | 0.07 | 0.00 |
| 25–29 | 0.00 | 0.96 | 0.96 | 43.59 | 0.96 |  | 0.00 | 0.00 | 0.00 | 0.07 | 0.00 |
| 30–34 | 0.11 | 1.14 | 1.25 | 57.02 | 1.26 |  | 0.00 | 0.00 | 0.00 | 0.07 | 0.00 |
| 35–39 | 0.03 | 0.64 | 0.67 | 30.49 | 0.67 |  | 0.00 | 0.00 | 0.00 | 0.07 | 0.00 |
| 40–44 | 0.05 | 0.98 | 1.03 | 46.78 | 1.03 |  | 0.00 | 0.38 | 0.38 | 13.08 | 0.39 |
| 45–49 | 0.09 | 0.71 | 0.79 | 36.19 | 0.80 |  | 0.04 | 0.12 | 0.17 | 5.64 | 0.17 |
| 50–54 | 0.03 | 0.24 | 0.26 | 11.86 | 0.26 |  | -0.03 | 0.18 | 0.16 | 5.41 | 0.16 |
| 55–59 | 0.02 | 0.32 | 0.34 | 15.51 | 0.34 |  | 0.05 | 0.48 | 0.54 | 18.34 | 0.54 |
| 60–64 | 0.05 | 0.17 | 0.22 | 9.83 | 0.22 |  | 0.02 | 0.54 | 0.57 | 19.37 | 0.57 |
| 65–69 | -0.01 | -0.03 | -0.04 | -1.79 | -0.04 |  | 0.07 | 0.39 | 0.46 | 15.71 | 0.46 |
| 70–74 | 0.00 | -0.24 | -0.24 | -10.81 | -0.24 |  | 0.01 | 0.51 | 0.51 | 17.63 | 0.52 |
| 75–79 | -0.07 | 0.09 | 0.01 | 0.59 | 0.01 |  | 0.10 | 0.67 | 0.77 | 26.45 | 0.78 |
| 80–84 | 0.11 | -0.75 | -0.64 | -29.22 | -0.65 |  | 0.07 | -0.57 | -0.51 | -17.35 | -0.51 |
| ≥ 85 | -0.25 | 0.00 | -0.25 | -11.55 | -0.26 |  | -0.53 | 0.00 | -0.53 | -18.20 | -0.54 |
| Total | 0.04 | 2.15 | 2.19 | 100.00 | 2.21 |  | -0.16 | 3.08 | 2.92 | 100.00 | 2.96 |

LE, life expectancy.

Gender gap, calculated as the LE of females minus that of males within each age group.

* By applying Arriaga's decomposition method.
