# Supplementary material for: Life expectancy and healthy life expectancy of patients with advanced schistosomiasis in Hunan Province, China
Source: Infect Dis Poverty. 2023 Jan 28;12:4. doi: 10.1186/s40249-023-01053-8 (PMC9883924; doi:10.1186/s40249-023-01053-8)
Supplement: Supplementary file 4 — Additional file 4: Age-specific contributions to the changes in life expectancy decomposed by Pollard’s decomposition method. [file 40249_2023_1053_MOESM4_ESM.docx]

**Age-specific contributions to the changes in life expectancy decomposed by Pollard’s decomposition method**

**1. Pollard’s decomposition method**

Same as the Arriaga's method, the Pollard’s decomposition method[1,2] was calculated to examine the difference in life expectancy (LE) between (i) general population and patients with advanced schistosomiasis and (ii) males and females in patients and general group, respectively. This is given by the formula:

$${}^{2-1}=e_{15}^{2}-e_{15}^{1}=\sum_{x=15}^{w} \left( {}_{n}{Q_{x}^{1}}-{}_{n}{Q_{x}^{2}} \right)\times{}_{x};with {}_{n}{Q_{x}}=-ln\left( l_{x+n}/l_{x} \right)$$

$${}_{x}=\frac{1}{2}\left( {}_{x}{P_{15}^{2}e_{x}^{1}}+{}_{x}{P_{15}^{1}e_{x}^{2}} \right); with {xP}_{15}=l_{x}/l_{15}$$

where *e_x_^1^* and *e_x_^2^* are the LEs at age *x* in group 1 (general population or males) and group 2 (patients with schistosomiasis or females); *w* is the last included age interval; *n* is the age interval which is 5 in this study; *_n_Q_x_* reflects the mortality rate at age *x* with weight *ω_x_*; (*_n_Q_x_^1^*-*_n_Q_x_^2^*)*ω_x_* gives the weight of each age group in the difference observed between the two LEs; *_x_P_15_* denotes the probability of surviving from age 15 to age *x*; and *l_x_* and *l_x+n_* is the number of individuals alive at age *x* and age *x*+*n*.

**2. Gaps in life expectancy**


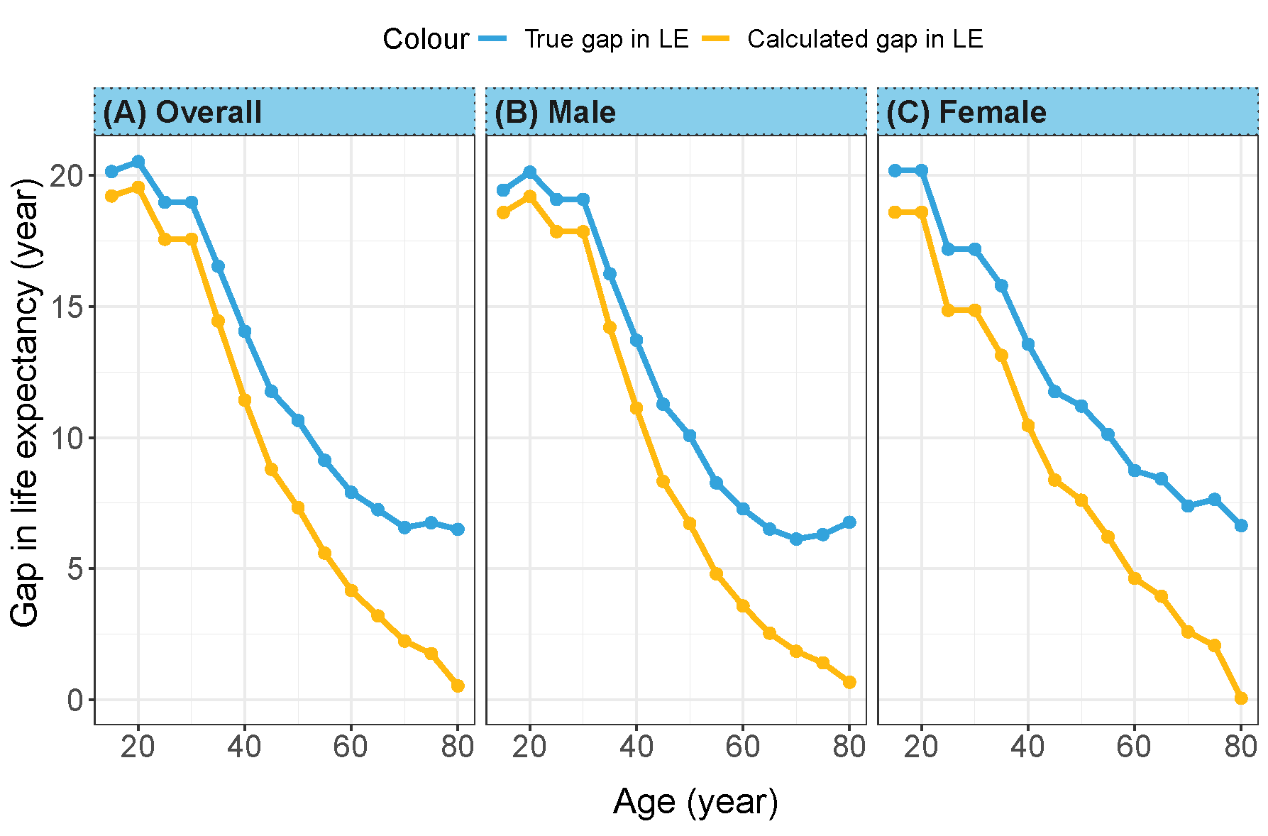


**Gap in life expectancy between advanced schistosomiasis patients and general population.** The true gap in LE is calculated by traditional life table, and the calculated gap in LE is estimated by Pollard’ method. The gap in LE (years) is the absolute value of difference in LE between patients with advanced schistosomiasis and general population. (A) Gap in LE between two groups in overall. (B) Gap in LE between two groups in males only. (C) Gap in LE between two groups in females only.

LE: life expectancy.

The difference in LEs (for overall subjects, males and females) between patients with advanced schistosomiasis and general population are underestimated by Pollard’s decomposition method, and the gap becomes widen with age increasing when compared to the true values of gap in LE.


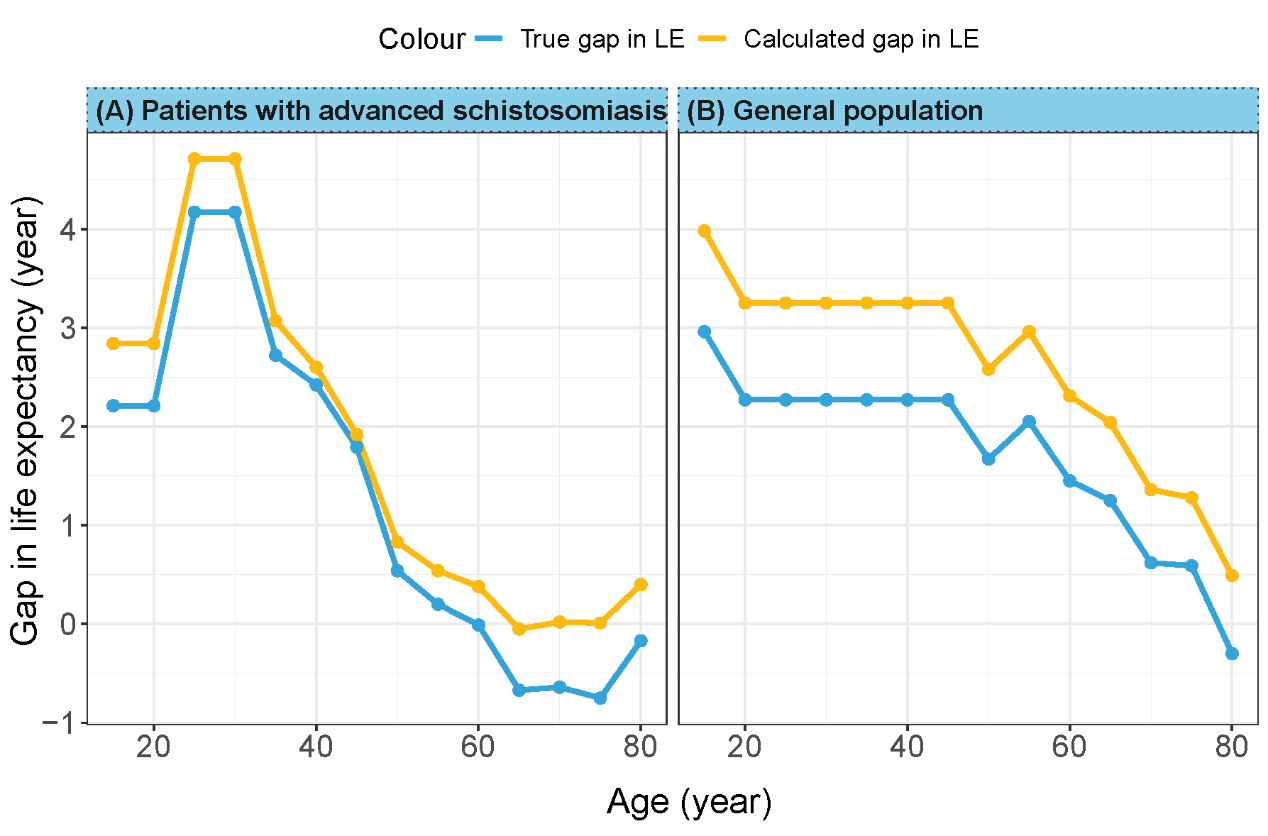


**Gender gap in life expectancy in advanced schistosomiasis patients and general population.** The true gap in LE is calculated by traditional life table, and the calculated gap in LE is estimated by Pollard’ method. The gender gap in LE (years) is calculated as the LE of females minus that of males within each age group. (A) Gender gap in LE in patients with advanced schistosomiasis. (B) Gender gap in LE in general population.

LE: life expectancy.

The trend of the gender gap in LE calculated by Pollard’s decomposition method is similar to the true gender gap; while the difference in LEs between males and females in both patients with advanced schistosomiasis and general population are overestimated.

**3. Age-specific mortality contributions**

**Decomposition of the age-specific mortality contribution to the changes in life expectancy in patients with advanced schistosomiasis and general population**

| Age group* | Gap in overall LE between patients and general group | | Gap in female LE between patients and general group | | Gap in male LE between patients and general group | | Gap in LE between females and males in patients (a) | | Gap in LE between females and males in general group (b) | | Changes in gender gap of LE between patients and general group (b-a) | |
| --- | --- | --- | --- | --- | --- | --- | --- | --- | --- | --- | --- | --- |
|  | Years | % | Years | % | Years | % | Years | % | Years | % | Years | % |
| 15–19 | 0.34 | -1.75 | 0.62 | -3.31 | 0.00 | 0.00 | 0.00 | 0.00 | 0.73 | 18.42 | 0.73 | 64.19 |
| 20–24 | -1.98 | 10.33 | -1.34 | 7.21 | -3.74 | 20.12 | -1.87 | -65.97 | 0.00 | 0.00 | 1.87 | 163.99 |
| 25–29 | 0.00 | 0.00 | 0.00 | 0.00 | 0.00 | 0.00 | 0.00 | 0.00 | 0.00 | 0.00 | 0.00 | 0.00 |
| 30–34 | -3.12 | 16.23 | -3.65 | 19.67 | -1.72 | 9.23 | 1.64 | 57.87 | 0.00 | 0.00 | -1.64 | -143.85 |
| 35–39 | -3.01 | 15.69 | -3.08 | 16.55 | -2.67 | 14.38 | 0.46 | 16.34 | 0.00 | 0.00 | -0.46 | -40.62 |
| 40–44 | -2.64 | 13.73 | -2.79 | 15.01 | -2.08 | 11.20 | 0.68 | 24.13 | 0.00 | 0.00 | -0.68 | -59.99 |
| 45–49 | -1.46 | 7.62 | -1.61 | 8.69 | -0.76 | 4.10 | 1.09 | 38.39 | 0.67 | 16.86 | -0.42 | -36.66 |
| 50–54 | -1.74 | 9.07 | -1.92 | 10.35 | -1.41 | 7.58 | 0.29 | 10.24 | -0.38 | -9.58 | -0.67 | -58.85 |
| 55–59 | -1.41 | 7.35 | -1.21 | 6.54 | -1.58 | 8.51 | 0.16 | 5.74 | 0.65 | 16.30 | 0.49 | 42.56 |
| 60–64 | -0.97 | 5.05 | -1.04 | 5.61 | -0.68 | 3.65 | 0.43 | 15.18 | 0.27 | 6.66 | -0.17 | -14.52 |
| 65–69 | -0.97 | 5.02 | -0.69 | 3.73 | -1.35 | 7.28 | -0.07 | -2.45 | 0.69 | 17.23 | 0.76 | 66.15 |
| 70–74 | -0.48 | 2.50 | -0.44 | 2.37 | -0.53 | 2.84 | 0.01 | 0.19 | 0.08 | 1.92 | 0.07 | 6.22 |
| 75–79 | -1.23 | 6.41 | -0.73 | 3.94 | -2.00 | 10.78 | -0.39 | -13.81 | 0.79 | 19.93 | 1.19 | 103.80 |
| 80–84 | -0.53 | 2.75 | -0.67 | 3.63 | -0.06 | 0.33 | 0.40 | 14.15 | 0.49 | 12.26 | 0.09 | 7.56 |
| **Total** | **-19.21** | **100.00** | **-18.58** | **100.00** | **-18.59** | **100.00** | **2.84** | **100.00** | **3.98** | **100.00** | **1.14** | **100.00** |

Abbreviation: LE, life expectancy.

The of age-specific mortality contribution was decomposed by Pollard’s method.

*The contribution of age group of 85 above (the open-ended age interval) cannot be calculated.

**(A) (B)**


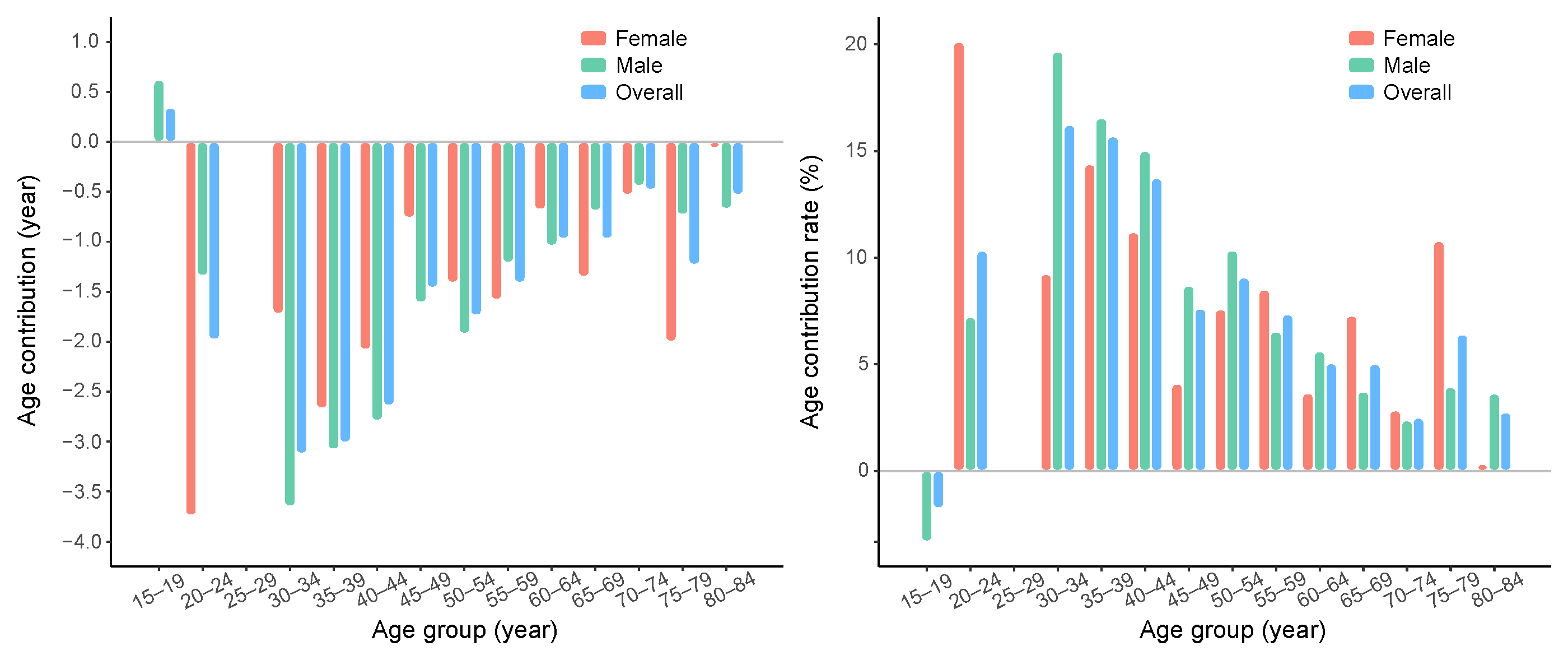


**Age-specific mortality contribution to the gap in LE between advanced schistosomiasis patients and general population.** (A) Age-specific mortality contribution to the gap in LE (years); (B) The rate of age-specific mortality contribution to the gap in LE. The age-specific mortality contribution was decomposed by Pollard’s method.

LE: life expectancy.

**(A) (B)**


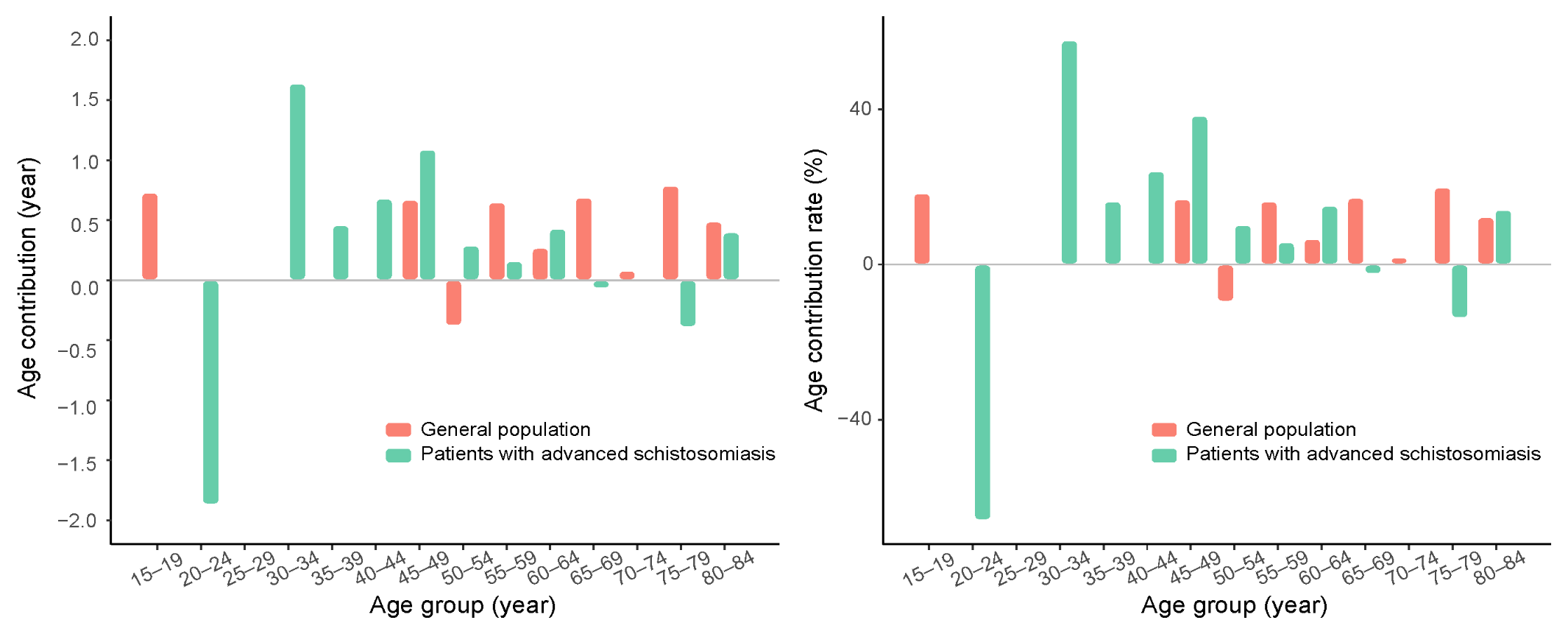


**Age-specific mortality contribution to the gender gap in LE in advanced schistosomiasis patients and general population.** (A) Age-specific mortality contribution to the gap in LE (years); (B) The rate of age-specific mortality contribution to the gap in LE. The age-specific mortality contribution was decomposed by Pollard’s method.

LE: life expectancy.

**4. Discussion**

In this study, the difference in true gap in LE and the gap calculated by Pollard’s method was found to be widen as age increased. The Pollard’s decomposition method adopts addition approximation integral of mortality in the small age-range (i.e., a 5-year interval)[1]. The gap in LE (*Δ^2-1^*) is calculated by assuming that the weight *ω_x_* is almost linear over most of the age range (as *_x_P_0_^2^e_x_^1^* and *_x_P_0_^1^e_x_^2^* are of comparable magnitude) and merging the relatively small interaction terms with the main effects in the formula[1]. This hypothesis can lead to residual effects in estimating the LE at a specific age[3].

The different contribution patterns decomposed by Pollard’s method from Arriaga’s method especially in advanced age groups might be explained by the relatively high mortality rate at age 80 and above in our patients. Since the contribution of the last open-ended age interval (85+ years) cannot be calculated, the estimated error could be large if the mortality rate of this age group is high[4]. In addition, when the analysis extended to include cause of death (i.e., advanced schistosomiasis in this study), the contribution of the mortality changes common to both sexes to the change in LE differential can be thought of as an interaction effect, which is combined in the main part of the formula[2]. It may reduce the precision of the decomposition and lead to a more difficult interpretation of the results.

**References**

1. Pollard JH. The expectation of life and its relationship to mortality. J Institut Actuaries. 1982;109(2):225-40.

2. Pollard JH. On the decomposition of changes in expectation of life and differentials in life expectancy. Demography. 1988;25(2):265-76.

3. Peng F, Feng T. The Comparison of Two Decomposition Methods on Life Expectancy Differences. Population Research. 2011;35(3):97-105 (**in Chinese**).

4. Peng F, Wanner P. Decomposition Model on Life Expectancy Differences and its Application. Chinese Journal of Health Statistics. 2006; 6:536-39 (**in Chinese**).
