# Supplementary material for: Life expectancy and healthy life expectancy of patients with advanced schistosomiasis in Hunan Province, China
Source: Infect Dis Poverty. 2023 Jan 28;12:4. doi: 10.1186/s40249-023-01053-8 (PMC9883924; doi:10.1186/s40249-023-01053-8)
Supplement: Supplementary file 5 — Additional file 5: Healthy life expectancy and its gender gap among patients with advanced schistosomiasis. [file 40249_2023_1053_MOESM5_ESM.docx]

**Healthy life expectancy and its gender gap among patients with advanced schistosomiasis**

| **Age group** | **Disabled weight** | **Total population** | | | |  | **Male** | | | |  | **Female** | | | | **Gender gap (F-M)** |
| --- | --- | --- | --- | --- | --- | --- | --- | --- | --- | --- | --- | --- | --- | --- | --- | --- |
|  |  | **YDL** | **HLE loss** | **HLE** | **HLE left** |  | **YDL** | **HLE loss** | **HLE** | **HLE left** |  | **YDL** | **HLE loss** | **HLE** | **HLE left** |  |
| 15–19 | 0.378 | 23.55 | 18.72 | 30.80 | 3.11 |  | 19.39 | 18.47 | 30.39 | 3.11 |  | 4.12 | 19.30 | 31.76 | 3.11 | 1.38 |
| 20–24 | 0.378 | 104.10 | 16.83 | 27.69 | 2.14 |  | 75.71 | 16.58 | 27.28 | 2.46 |  | 28.39 | 17.41 | 28.65 | 1.24 | 1.38 |
| 25–29 | 0.378 | 176.83 | 15.52 | 25.54 | 3.11 |  | 122.21 | 15.08 | 24.82 | 3.11 |  | 54.62 | 16.66 | 27.41 | 3.11 | 2.59 |
| 30–34 | 0.378 | 512.45 | 13.63 | 22.43 | 1.59 |  | 360.91 | 13.19 | 21.71 | 1.35 |  | 151.54 | 14.77 | 24.30 | 2.24 | 2.59 |
| 35–39 | 0.378 | 1449.44 | 12.66 | 20.84 | 1.57 |  | 1102.85 | 12.37 | 20.36 | 1.53 |  | 346.59 | 13.41 | 22.06 | 1.72 | 1.70 |
| 40–44 | 0.378 | 2595.46 | 11.71 | 19.27 | 2.28 |  | 1911.58 | 11.44 | 18.83 | 2.18 |  | 683.88 | 12.36 | 20.34 | 2.61 | 1.51 |
| 45–49 | 0.399 | 2851.57 | 11.28 | 16.99 | 1.89 |  | 1996.92 | 11.06 | 16.65 | 1.68 |  | 854.70 | 11.77 | 17.73 | 2.43 | 1.08 |
| 50–54 | 0.399 | 4063.34 | 10.02 | 15.10 | 1.87 |  | 2876.51 | 9.94 | 14.98 | 1.81 |  | 1186.83 | 10.16 | 15.30 | 2.02 | 0.33 |
| 55–59 | 0.399 | 5672.42 | 8.78 | 13.22 | 4.04 |  | 3858.21 | 8.74 | 13.16 | 4.00 |  | 1814.21 | 8.82 | 13.28 | 4.12 | 0.12 |
| 60–64 | 0.510 | 6945.59 | 9.56 | 9.18 | 1.62 |  | 4682.36 | 9.54 | 9.16 | 1.52 |  | 2263.28 | 9.53 | 9.16 | 1.84 | -0.01 |
| 65–69 | 0.510 | 5846.90 | 7.87 | 7.57 | 1.66 |  | 4194.39 | 7.96 | 7.65 | 1.66 |  | 1652.55 | 7.62 | 7.32 | 1.65 | -0.33 |
| 70–74 | 0.510 | 3805.31 | 6.15 | 5.91 | 1.68 |  | 2824.33 | 6.23 | 5.99 | 1.67 |  | 980.93 | 5.91 | 5.67 | 1.72 | -0.31 |
| 75–79 | 0.510 | 1275.20 | 4.40 | 4.23 | 1.18 |  | 1015.16 | 4.49 | 4.32 | 1.30 |  | 305.95 | 4.11 | 3.95 | 1.02 | -0.37 |
| 80–84 | 0.510 | 342.98 | 3.17 | 3.05 | 1.47 |  | 233.53 | 3.14 | 3.02 | 1.28 |  | 109.45 | 3.05 | 2.93 | 1.89 | -0.09 |
| ≥ 85 | 0.510 | 36.16 | 1.64 | 1.58 | 1.58 |  | 30.75 | 1.81 | 1.74 | 1.74 |  | 5.41 | 1.08 | 1.04 | 1.04 | -0.70 |

HLE, healthy life expectancy; YDL, years lived with disability.

Gender gap, calculated as the LE of females minus that of males within each age group.
